# Description of Short-Range Interactions of Carbon-Based Materials with a Combined AIREBO and ZBL Potential

**DOI:** 10.3390/nano14171423

**Published:** 2024-08-31

**Authors:** Jing Li, Tan Shi, Yichao Sun, Xintian Cai, Rui Gao, Qing Peng, Peng Lu, Chenyang Lu

**Affiliations:** 1 School of Nuclear Science and Technology, Xi’an Jiaotong University, Xi’an 710049, China; 4120103221@stu.xjtu.edu.cn (J.L.); ruigao@xjtu.edu.cn (R.G.); 2 Institute of Microelectronics, Chinese Academy of Sciences, Beijing 100029, China; sunyichao@ime.ac.cn (Y.S.); lupeng@ime.ac.cn (P.L.); 3 School of Integrated Circuits, University of Chinese Academy of Sciences, Beijing 100049, China; 4 Key Laboratory of Science and Technology on Silicon Devices, Chinese Academy of Sciences, Beijing 100029, China; 5 State Key Laboratory of Nonlinear Mechanics, Institute of Mechanics, Chinese Academy of Sciences, Beijing 100190, China; caixintian@whu.edu.cn; 6 Center of Materials Science and Optoelectronics Engineering, University of Chinese Academy of Sciences, Beijing 100049, China; 7 Guangdong Aerospace Research Academy, Guangzhou 511458, China; 8 State Key Laboratory of Multiphase Flow in Power Engineering, Xi’an Jiaotong University, Xi’an 710049, China

**Keywords:** ion irradiation, irradiation damage, carbon, graphene, molecular dynamics

## Abstract

An accurate description of short-range interactions among atoms is crucial for simulating irradiation effects in applications related to ion modification techniques. A smooth integration of the Ziegler–Biersack–Littmark (ZBL) potential with the adaptive intermolecular reactive empirical bond-order (AIREBO) potential was achieved to accurately describe the short-range interactions for carbon-based materials. The influence of the ZBL connection on potential energy, force, and various AIREBO components, including reactive empirical bond-order (REBO), Lennard–Jones (LJ), and the torsional component, was examined with configurations of the dimer structure, tetrahedron structure, and monolayer graphene. The REBO component is primarily responsible for the repulsive force, while the LJ component is mainly active in long-range interactions. It is shown that under certain conditions, the torsional energy can lead to a strong repulsive force at short range. Molecular dynamics simulations were performed to study the collision process in configurations of the C-C dimer and bulk graphite. Cascade collisions in graphite with kinetic energies of 1 keV and 10 keV for primary knock-on atoms showed that the short-range description can greatly impact the number of generated defects and their morphology.

## 1. Introduction

The ion irradiation of carbon-based materials, such as graphene [1,2,3,4,5,6,7,8,9], fullerene [10,11], carbon nanotubes [12,13,14], and graphite [15,16,17], has been widely studied in recent decades. The motivations and potential applications encompass doping [18,19], nanopore generation [5], nanopatterning [20], tuning of electronic properties [21], and irradiation damage assessment [1,22], among others. The incident particles include a wide range of ions, such as proton [1], helium [19], silicon [23], and argon [13], with incident kinetic energies ranging from ∼keV to ∼GeV. One significant physical process during ion irradiation is the displacement cascade induced by the energy transfer from the incident ion, primarily responsible for the generation of structural defects in the regime of high nuclear stopping power. Molecular dynamics (MD) simulations serve as a powerful tool for investigating the ion collision process in carbon-based materials, encompassing defect generation, annihilation, migration, and structural modifications. Most classical interatomic potentials focus on the description of atomic interactions at near-equilibrium distances. However, depending on the particle species and energies, ion irradiation can lead to much closer interatomic distances, necessitating an accurate description of short-range interactions.

This energy regime is predominantly dictated by the screened Coulomb interaction, which can be described by the Ziegler–Biersack–Littmark (ZBL) potential [24]. As a prerequisite for performing cascade collision simulations, different potentials need to be smoothly connected with the ZBL potential to accurately model the short-range interactions [1,25,26]. This integration typically involves the use of a smooth, distance-dependent weighting function—like a Fermi function or a polynomial function—to ensure a gradual transition between potentials. For carbon-based materials, the adaptive intermolecular reactive empirical bond-order (AIREBO) potential is a widely used potential which can accurately describe both the chemical processes and long-range intermolecular interactions in hydrocarbon systems [27]. The integration of the ZBL potential is also necessary when examining ion irradiation effects using the AIREBO potential. There are three components in the AIREBO potential: the reactive empirical bond-order (REBO) component, the Lennard–Jones (LJ) component, and the torsional component. These components dominate or vanish under different conditions based on the local chemical environment and the corresponding interatomic distances and bond orders. In addition, unlike some other potentials with weak short-range interactions, the original repulsive force in AIREBO can be orders of magnitude stronger than the ZBL potential under certain circumstances [22], posing challenges for the adjustment of the potential function. Given these complexities, a smooth connection with the ZBL potential is not trivial for the AIREBO potential.

For most of the MD studies on the ion irradiation of carbon-based materials, the ZBL potential is only used to describe the interactions between the incident ion and carbon atoms, leaving the AIREBO potential unmodified for carbon atoms [5,6,7,10,13,22,28,29,30,31,32,33,34,35]. This approach is only deemed acceptable when the energy transferred to the carbon atoms is low or when high-energy carbon atoms move in a direction that precludes further collisions. To provide a quantitative view, calculations from this work showed that a head-on collision involving a 1 keV carbon atom can achieve a C-C distance of less than 0.3 Å, which is far below the equilibrium distance between carbon atoms. In low-dimensional materials, such as monolayer graphene or carbon nanotube, although interlayer collisions are nonexistent or greatly reduced, cascade collisions along the carbon plane can still occur. Therefore, incorporating the ZBL interaction is crucial for accurately modeling ion interactions, especially when the energy transfer from the incident ion is substantial. In previous MD simulations, the use of the AIREBO potential along with the ZBL potential has been achieved by several studies through various means [21,36,37,38,39,39]. However, the methodology and the resulting effect on the original AIREBO potential are not extensively explained. We note that if the ZBL potential is only connected to the pair portion of the REBO potential without reducing the weight of other energy and force components, then the overall effect may not actually approach the ZBL interaction at short range.

In this study, the AIREBO potential is connected by a Fermi-like function for the carbon systems, and the resulting effects on different energy components are critically evaluated. Our results show that the ability to achieve a smooth potential connection depends on the specific chemical environment. It is therefore crucial to understand the impact of ZBL inclusion on the atomic interactions across various chemical environments for accurate modeling of ion interactions and interpretation of the results. The methodology presented in this work is also relevant for irradiation damage studies for other graphene-like two-dimensional materials [40,41,42] using similar potential forms [43,44,45].

## 2. Materials and Methods

### 2.1. Interatomic Potential Description

All of the simulations were performed using the Large-Scale Atomic/ Molecular Massively Parallel Simulator (LAMMPS) code [46]. The AIREBO potential has the form

(1) $$E^{\mathrm{AIREBO}}=E^{\mathrm{REBO}}+E^{\mathrm{LJ}}+E^{\mathrm{tors}},$$

where $E^{\mathrm{REBO}}$, $E^{\mathrm{LJ}}$, and $E^{\mathrm{tors}}$ are the REBO energy, LJ energy, and torsional energy, respectively. The REBO potential is used to describe covalent bonding interactions, while the LJ potential complements the REBO potential in describing dispersion and long-range interactions. The torsional component provides a more accurate description of torsional interactions, enhancing the modeling of large hydrocarbon structures [27]. A comprehensive explanation of these energy terms is provided in the original publication [27], and a concise overview is presented here to explain the factors influencing these energy components. The REBO energy between atom *i* and *j* is the sum of two components:

(2) $$E_{ij}^{\mathrm{REBO}}=V_{ij}^{R}\left(r_{ij}\right)+b_{ij}V_{ij}^{A}\left(r_{ij}\right),$$

where $V_{ij}^{R}\left(r_{ij}\right)$ is a pairwise repulsive potential, and $b_{ij}V_{ij}^{A}\left(r_{ij}\right)$ is a pairwise attractive potential multiplied by a many-body bond-order term. The bond order $b_{ij}$ takes into account the strength of covalent bonding based on the local chemical environment. The LJ term is included in the AIREBO potential to account for the intermolecular interactions. It is built on the original LJ potential, $V_{ij}^{\mathrm{LJ}}\left(r_{ij}\right)$, and incorporates three cutoff and switching functions: $S(t_{b}\left(b_{ij}^{*}\right))$, $C_{ij}$, and $S(t_{r}\left(r_{ij}\right))$. The expression is as follows:

(3) $$\begin{array}{cc} E_{ij}^{\mathrm{LJ}}= & S\left(t_{r}\left(r_{ij}\right)\right)S\left(t_{b}\left(b_{ij}^{*}\right)\right)C_{ij}V^{\mathrm{LJ}}\left(r_{ij}\right)+\\ \; & \left[1-S\left(t_{r}\left(r_{ij}\right)\right)\right]C_{ij}V_{ij}^{\mathrm{LJ}}\left(r_{ij}\right), \end{array}$$

where t(x) is a function to rescale the variable *x*, and S(t) is a general-purpose smooth switching function. The $S(t_{b}\left(b_{ij}^{*}\right))$ term is a bond order-dependent switching function used to reduce the strength of LJ energy when the bond order is large, where $b_{ij}^{*}$ is a modified expression of the original bond order $b_{ij}$ used to accurately describe the atomic interactions at intermolecular distances. The $C_{ij}$ term is used to completely or partially disable LJ energy for bonded or partially bonded configurations. This term involves a series of distance-dependent weighting parameters used to characterize the extent of bonding based on the neighboring environment. The $S(t_{r}\left(r_{ij}\right))$ term is a distance-dependent switching function used to reduce the strength of LJ energy at shorter distances. It works together with $S(t_{b}\left(b_{ij}^{*}\right))$ and $C_{ij}$ to collectively determine the weight of the original LJ energy. The torsional energy takes into account the torsional interactions based on the dihedral angle ω:

(4) $$E_{ij}^{\mathrm{tors}}=\sum_{k\neq i,j}\sum_{l\neq i,j,k}w_{ij}\left(r_{ij}\right)w_{jk}\left(r_{jk}\right)w_{kl}\left(r_{kl}\right)\times V^{\mathrm{tors}}\left(\omega_{ijkl}\right),$$

where *w* is a distance-dependent weighting function, and $V_{\mathrm{tors}}$ is a torsional potential that depends on the dihedral angle.

The pairwise ZBL potential is expressed as [24]

(5) $$\begin{array}{cc} E_{ij}^{\mathrm{ZBL}}\left(r_{ij}\right) & =\frac{1}{4\pi \epsilon_{0}}\frac{Z_{i}Z_{j}\;e^{2}}{r_{ij}}\phi \left(r_{ij}/a\right), \end{array}$$

(6) $$\begin{array}{cc} a & =\frac{0.8854\;a_{0}}{Z_{i}^{0.23}+Z_{j}^{0.23}}, \end{array}$$

(7) $$\begin{array}{cc} \phi (r_{ij}/a) & =\sum_{m}c_{m}e^{-f_{m}\frac{r_{ij}}{a}}, \end{array}$$

where $\epsilon_{0}$ is the vacuum permittivity, *Z* is the atomic number, *e* is the electron charge, $a_{0}$ is the Bohr radius, and $c_{m}$ and $f_{m}$ are coefficient sets to describe the screened Coulomb interactions.

### 2.2. Interatomic Potential Integration

The connection between the AIREBO and ZBL potential was achieved by a Fermi-like switching function:

(8) $$\begin{array}{c} f_{F}\left(r\right)=\frac{1}{1+e^{-A_{F}(r-r_{C}})}. \end{array}$$

Here, $f_{F}\left(r\right)$ varies smoothly from ∼0 to 1 with the increase of distance and is equal to 0.5 when $r=r_{c}$. $A_{F}$ controls the sharpness of the transition. The energy of the combined potential is expressed as

(9) $$\begin{array}{cc} E_{ij}^{\mathrm{combined}}= & \left(1-f_{F}\left(r_{ij}\right)\right)E_{ij}^{\mathrm{ZBL}}\left(r_{ij}\right)+\\ \; & f_{F}\left(r_{ij}\right)E_{ij}^{\mathrm{AIREBO}}. \end{array}$$

We note that $E_{ij}^{\mathrm{ZBL}}\left(r_{ij}\right)$ and $f_{F}\left(r_{ij}\right)$ depend only on $r_{ij}$, whereas $E_{ij}^{\mathrm{AIREBO}}$ depends on multiple variables determined by the local chemical environment, including the bond order, the position vectors of other atoms involved in the torsional interactions, etc. Here, the switching function needs to be applied to the entire $E^{\mathrm{AIREBO}}$ in order to dampen all of the AIREBO energy terms. In the actual implementation, the ZBL term was grouped with the REBO term:

(10) $$\begin{array}{cc} E_{ij}^{\mathrm{combined}}= & [\left(1-f_{F}\left(r_{ij}\right)\right)E_{ij}^{\mathrm{ZBL}}\left(r_{ij}\right)+f_{F}\left(r_{ij}\right)E_{ij}^{\mathrm{REBO}}]+\\ \; & f_{F}\left(r_{ij}\right)E_{ij}^{\mathrm{LJ}}+f_{F}\left(r_{ij}\right)E_{ij}^{\mathrm{tors}}. \end{array}$$

For the energy component of $E_{ij}^{\mathrm{combined}}$ between atom *i* and atom *j*, the corresponding force components of atom *i* are

(11) $$\begin{array}{ccc} {\vec{F}}_{ij,E_{ij}^{\mathrm{combined}}} & =-\frac{\partial E_{ij}^{\mathrm{combined}}}{\partial {\vec{r}}_{ij}},{\vec{F}}_{ik,E_{ij}^{\mathrm{combined}}} & =-\frac{\partial E_{ij}^{\mathrm{combined}}}{\partial {\vec{r}}_{ik}}. \end{array}$$

We note that if $r_{ik}$ is involved in $E_{ij}^{\mathrm{combined}}$, it can also lead to a force component in atom *i*. The only difference during the gradient operation is that the switching function and the ZBL term in $E_{ij}^{\mathrm{combined}}$ are independent of $r_{k}$. Overall, the partial derivatives or gradient operations shown in Equation (11) need to take into account all of the terms presented in the listed equations, such as $f_{F}\left(r_{ij}\right)$, $E^{\mathrm{ZBL}}\left(r_{ij}\right)$, $S(t_{b}\left(b_{ij}^{*}\right))$, $C_{ij}$, etc. In the implementation, the forces related to ZBL were also grouped with the REBO portion, whereas other terms were only influenced by the switching function $f_{F}$. For instance, the forces associated with the modified REBO and LJ terms were defined as

(12) $$\begin{array}{cc} {\vec{F}}_{ij,E_{ij}^{\mathrm{combined}}}^{\;\mathrm{REBO}+\mathrm{ZBL}}= & -\frac{f_{F}\left({\vec{r}}_{ij}\right)\partial E_{ij}^{\mathrm{REBO}}+\left(1-f_{F}\left({\vec{r}}_{ij}\right)\right)\partial E_{ij}^{\mathrm{ZBL}}}{\partial {\vec{r}}_{ij}}\\ \; & -\frac{\left(E_{ij}^{\mathrm{REBO}}-E_{ij}^{\mathrm{ZBL}}\right)df_{F}\left({\vec{r}}_{ij}\right)}{d{\vec{r}}_{ij}}, \end{array}$$

(13) $$\begin{array}{cc} {\vec{F}}_{ij,E_{ij}^{\mathrm{combined}}}^{\;\mathrm{LJ}}= & -\frac{f_{F}\left({\vec{r}}_{ij}\right)\partial E_{ij}^{\mathrm{LJ}}}{\partial {\vec{r}}_{ij}}-\frac{E_{ij}^{\mathrm{LJ}}df_{F}\left({\vec{r}}_{ij}\right)}{d{\vec{r}}_{ij}}. \end{array}$$

The other terms, including the torsional force, were calculated in a similar manner. Overall, the connection with the ZBL potential was achieved by applying a distance-dependent weighting function to the AIREBO potential, and force calculations were performed according to gradient operations considering various distance-containing terms.

### 2.3. Simulation Setup for Potential Comparison

**Figure 1:**
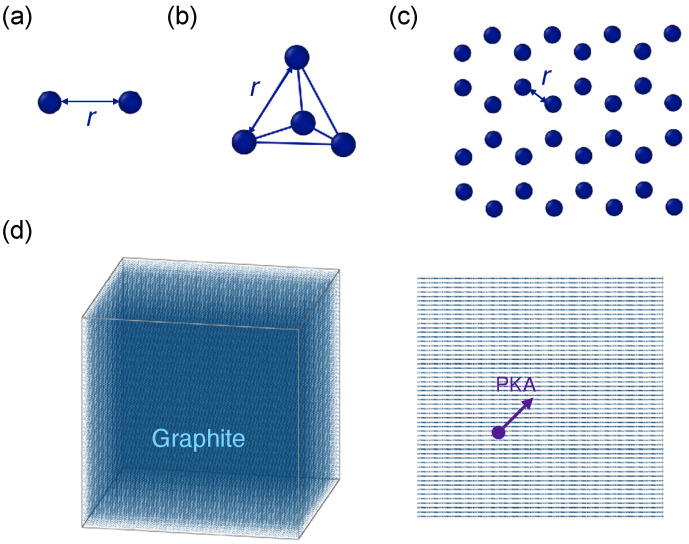
Simulation setups of (**a**) dimer configuration, (**b**) tetrahedron configuration, (**c**) graphene configuration, and (**d**) graphite configuration.

The combined potential (denoted as AIREBO-ZBL), the original AIREBO potential, and the ZBL potential were compared under several configurations. The total and partial potential energy and force components were determined as a function of the atomic distance. First, simulations of a dimer configuration consisting of two carbon atoms were conducted under fixed boundary conditions (see Figure 1a). Head-on collisions with the dimer configuration were also simulated by assigning different velocities to one of the carbon atoms. Subsequently, a tetrahedral configuration of four equidistant carbon atoms was examined (see Figure 1b). Next, monolayer graphene with periodic boundary conditions in the plane direction was simulated with varying lattice constants (see Figure 1c).

Finally, cascade collisions were performed in graphite. Graphite was chosen instead of graphene because it is likely for atoms to leave the graphene layer without producing substantial cascade collisions [1]. The simulation box had dimensions of 182 Å × 177 Å × 181 Å with a total of ∼680,000 atoms (see Figure 1d). The structure was initially optimized and equilibrated at 300 K with the NPT ensemble. Next, a primary knock-on atom (PKA) was imparted with a kinetic energy of 1 keV or 10 keV at a direction of 〈111〉. A variable timestep was used to accurately capture the short-range collision process. The timestep can vary from 10^−5^ fs to 1 fs with a maximum allowable displacement 0.1 Å/step and a maximum energy transfer of 10 eV/step. The displacement cascade process was then simulated using the NVE ensemble for 30–50 ps in order to ensure the stabilization of defect numbers. The structure was then relaxed under NVT ensemble for 10 ps. A final structural optimization through energy minimization was conducted for defect analysis. It was found that the Wigner–Seitz defect analysis was not reliable enough to characterize the displaced atoms due to the rippling and buckling of the graphite atomic layers. The number of atoms in the defective regions was determined based on the atomic positions and coordination numbers [47,48]. A first nearest-neighbor (1NN) cutoff of 1.7 Å and a 3NN cutoff of 3.0 Å were used to identify atoms that were not properly bonded. Atoms positioned between layers were also classified as defects. In total, ten separate runs were executed for each potential at each PKA energy.

## 3. Results and Discussion

**Figure 2:**
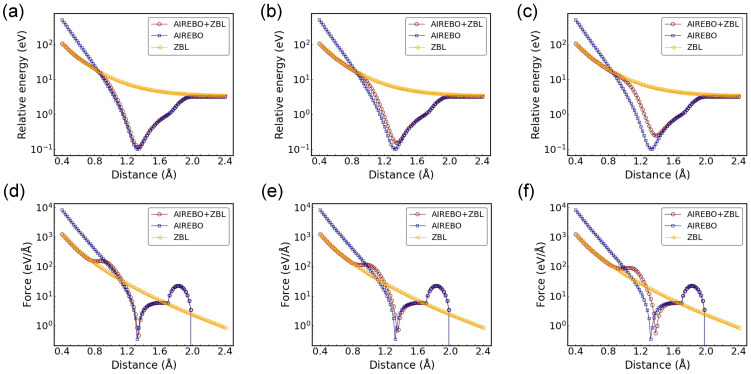
Potential energy and force as a function of C-C distance for the dimer configuration, with potential energy shown for (**a**) $r_{C}$ = 0.9 Å, (**b**) $r_{C}$ = 1.0 Å, and (**c**) $r_{C}$ = 1.1 Å and force shown for (**d**) $r_{C}$ = 0.9 Å, (**e**) $r_{C}$ = 1.0 Å, and (**f**) $r_{C}$ = 1.1 Å. The $A_{F}$ in the switching function is 14 ${Å}^{-1}$. The potential energy is shifted to positive values in the logarithmic plot.

First, the AIREBO-ZBL potential was compared with the AIREBO potential and the ZBL potential with a C-C dimer configuration. The parameters of the Fermi-like switching function need to be optimized so that the equilibrium distance of the studied structure remains largely unaffected, and the potential variation aligns with the ZBL at short-range distances. Furthermore, the variation in energy and force with interatomic distance needs to be as smooth as possible. In this dimer study, $A_{F}$ is set to 14 Å^−1^ in the switching function, and various switching distances ($r_{C}$) were investigated, as shown in Figure 2. As anticipated, the potential energy and force converge towards the ZBL potential at small C-C distances and align with the AIREBO potential at larger distances. In the dimer configuration, neither the LJ energy nor the torsional energy is activated, and the total energy and force are only contributed by the REBO component. The repulsive part of the REBO energy is stronger than the ZBL potential at short distances with a higher magnitude and slope. It can be seen from Figure 2a–c that the potential wells of AIREBO and AIREBO-ZBL coincide at $r_{C}$ = 0.9 Å. As $r_{C}$ increases, there are noticeable changes in both the potential well depth and the equilibrium distance. The shifts in equilibrium distance are approximately 0.02 Å and 0.06 Å for $r_{C}$ = 1.0 Å and $r_{C}$ = 1.1 Å, respectively. Concurrently, with the increase in $r_{C}$, the AIREBO-ZBL potential converges to the ZBL potential starting from a larger distance. It can be seen that the variation in $r_{C}$ affects the entire potential profile, influencing both equilibrium properties and short-range interactions. Regarding the force profiles (see Figure 2d–f), besides the similar observations made from the energy evolution, a bump appears between 0.8 Å and 1.1 Å. This increase in force largely originates from the $f_{F}^{\prime }E^{\mathrm{ZBL}}$ term.

**Figure 3:**
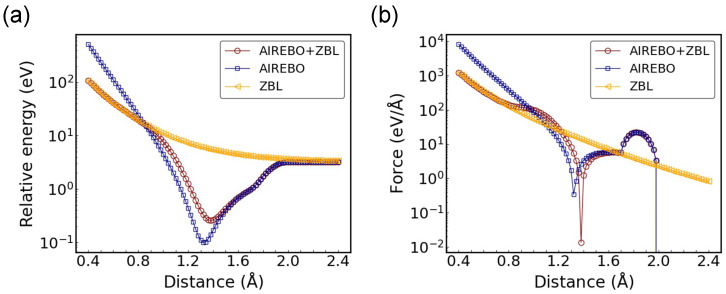
(**a**) Relative potential energy and (**b**) force as a function of C-C distance for the dimer configuration with $r_{C}$ = 1.0 Å and $A_{F}$ = 10 ${Å}^{-1}$.

With the same dimer configuration, reducing $A_{F}$ can lead to the attenuation of the bump magnitude but broadens its width in terms of distance. This is illustrated in Figure 3 with $r_{C}$ = 1.0 Å and $A_{F}$ = 10 ${Å}^{-1}$. In addition to the change in force, the energy profile is also modified compared to Figure 2b, indicating that a collective optimization of both $r_{C}$ and $A_{F}$ is essential. The presence of this small peak-like structure is unavoidable, and its shape needs to be balanced with considerations of the transition distance range and desired sharpness.

**Figure 4:**
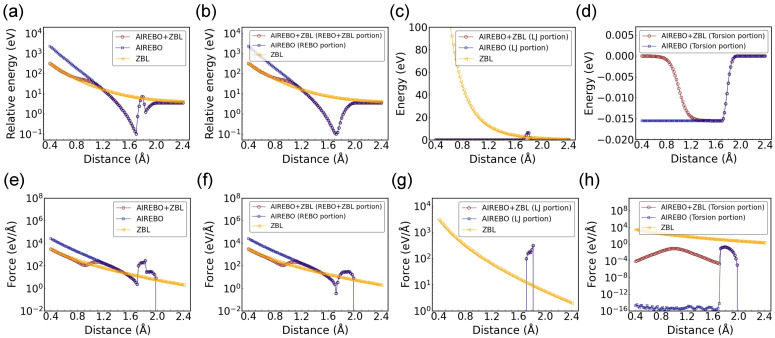
Potential energy and force as a function of C-C distance for the tetrahedron configuration with $r_{C}$ = 1.0 Å and $A_{F}$ = 14 ${Å}^{-1}$. The different energy and force components are shown for the AIREBO and AIREBO-ZBL potentials: (**a**) total energy, (**b**) REBO+ZBL/REBO energy, (**c**) LJ energy, (**d**) torsional energy; (**e**) total force, (**f**) REBO+ZBL/REBO force, (**g**) LJ force, and (**h**) torsional force. The potential energy is shifted to positive values in the logarithmic plot.

Since the dimer configuration does not include the torsional and LJ terms, a tetrahedron configuration with four equidistant carbon atoms was studied, with the corresponding energy and force components shown in Figure 4. This configuration was selected because the torsional energy requires a minimum of four atoms, and the same distance among all carbon atoms facilitates the interpretation of the potential energy and force variations with changing C-C distances. It is confirmed again that the total AIREBO-ZBL potential approaches the ZBL potential and AIREBO potential at the short-range and long-range ends, respectively. In this configuration, the REBO component still contributes a significant portion to the total energy and force, as seen by comparing Figure 4a,b and Figure 4e,f. The repulsive force from the REBO component is shown to be much stiffer than the screened Coulomb force from the ZBL potential at short range. As explained in the Section 2, the ZBL component was grouped with the REBO component. Thus, the agreement between AIREBO-ZBL and ZBL at short range is expected to be seen only in the REBO portion, but not in the LJ or torsional component.

Regarding the LJ component, it is deactivated at small distances and appears only between ∼1.72 Å and 1.82 Å (see Figure 4c,g). A good agreement between AIREBO-ZBL and AIREBO can be observed. The LJ energy in the AIREBO is primarily used to describe the intermolecular interactions at large distances, where the covalent bonding is not dominant [27]. The emergence of the LJ component within this narrow distance range can be attributed to the implementation of several switching functions in addition to the original LJ potential (see Equation (3)). We note that the repulsive portion of the LJ potential is extremely stiff (the $1/r^{12}$ term). If it were activated at short range, the switching function may not be sufficiently strong to align it perfectly with the ZBL potential at close proximities. This issue is not limited to the LJ potential. When there is a significant disparity between the studied potential and the ZBL potential, achieving a smooth connection between them without compromising their respective desired characteristics becomes difficult.

For the torsional portion in the tetrahedron configuration, as the C-C distance increases, the torsional energy in AIREBO-ZBL starts to deviate from zero at ∼0.8 Å and approaches that of the original AIREBO potential (see Figure 4d). It becomes fully aligned with the AIREBO potential at >1.2 Å. A noticeable difference in force is observed between 0.4 Å and 1.6 Å (see Figure 4h). This difference comes from the $f_{F}^{\prime }E^{\mathrm{tors}}$ term and is small compared to the ZBL component within this distance range. The absence of the smooth transition around $r_{C}$ = 1.0 Å is due to the cancellation of different torsional force components in the symmetric tetrahedron configuration. This was confirmed by examining different torsional force components among the four atoms along the *x*, *y*, and *z* directions.

**Figure 5:**
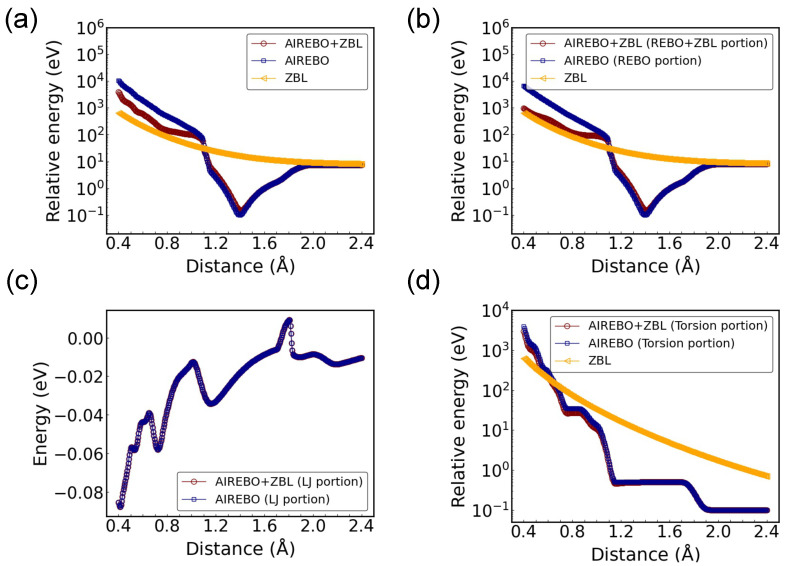
Potential energy and force as a function of the minimum C-C distance in monolayer graphene with $r_{C}$ = 1.0 Å and $A_{F}$ = 14 ${Å}^{-1}$. The different energy components are shown for the AIREBO and AIREBO-ZBL potentials: (**a**) total energy, (**b**) REBO+ZBL/REBO energy, (**c**) LJ energy, and (**d**) torsional energy. The potential energy is shifted to positive values in the logarithmic plot.

Subsequently, the configuration of monolayer graphene was studied with the AIREBO-ZBL potential. As presented in Figure 5a, the equilibrium C-C distance differs by ∼0.01 Å from the original AIREBO potential. The potential wells around the equilibrium position align closely, with a smaller disparity compared to the dimer configuration. However, the AIREBO-ZBL potential differs significantly from the ZBL potential at the short range end, in contrast to the observations in Figure 2 and Figure 4. By comparing the energy profiles between Figure 5a,b, it can be seen that the REBO energy significantly contributes to the total energy across most of the distance ranges. Compared to the total potential energy, there is a better agreement between the combined REBO + ZBL portion and ZBL at short range, with the difference being induced by the C-C atoms at larger distances. The total energy is not solely determined by the interactions of the closest C-C bonds, but is also affected by C-C interactions at farther distances. This effect also justifies the motivation to first conduct validation simulations with simplified configurations of equidistant C-C distances, circumventing the complexity introduced by the contributions from different neighboring distances. For the LJ energy, the complete agreement of the two energy profiles indicates that LJ interactions in graphene are long-ranged (see Figure 5c). Only C-C interactions from farther distances contribute to the LJ potential energy, which leads to the overlap of the energy profiles between the two potentials. The torsional energy of AIREBO-ZBL potential is expected to be greatly reduced at short distances. However, as shown in Figure 5d, an unexpectedly strong repulsive potential, surpassing the ZBL potential, is observed. Therefore, the disparity between AIREBO-ZBL and ZBL at short range in the total potential energy profile shown Figure 5a is primarily attributed to the unexpected torsional energy. Two factors must be considered here: First, the simulated configuration involves a varying lattice parameter that influences all the C-C distances. For ion irradiation simulations, it is unlikely for all atoms to have the same small distances. This configuration can therefore be considered as an extreme situation with respect to the ion irradiation scenarios. Second, Figure 5d indicates that the torsional energy is too strong to be suppressed at short range. This can result in a hard repulsive potential, even when the ZBL potential is smoothly connected. When the torsional term is included in the AIREBO, its behavior at small interatomic distances needs to be carefully examined for the simulation of different carbon systems.

**Figure 6:**
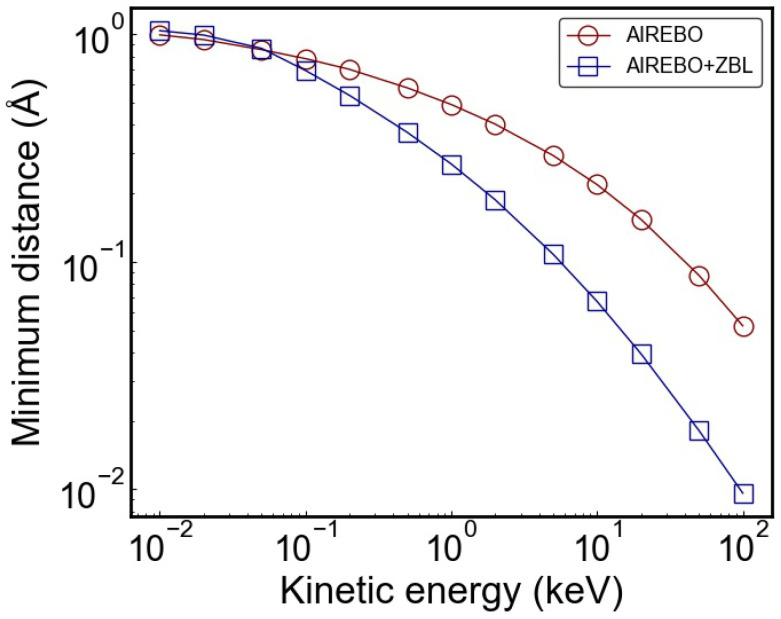
Comparison of the minimum distance between two carbon atoms in head-on collisions using the AIREBO and AIREBO-ZBL potentials.

Based on the aforementioned static energy calculations at varying C-C distances for different structural configurations, we confirmed the smooth integration of the ZBL potential with the AIREBO potential, with a detailed discussion on their effects on potential energy and force provided. Subsequently, dynamic simulations were performed with $r_{C}$ = 1.0 Å and $A_{F}$ = 14 ${Å}^{-1}$ in the switching function. It is noted that $r_{C}$ = 0.9 Å and slightly different $A_{F}$ values may also be suitable, depending on the specific requirements of the study. With the C-C dimer configuration, we varied the initial kinetic energies of one carbon atom in order to investigate the minimum distances achievable in head-on collisions, and the results are presented in Figure 6. Under the AIREBO-ZBL potential, the carbon atom can approach closer distances due to the softer repulsive interactions compared to the AIREBO potential. The differences between the two potentials are small for carbon kinetic energies below 100 eV but become more pronounced as the energy increases. For instance, when the carbon kinetic energies are 0.2 keV and 5 keV, the minimum distances are approximately 0.5 Å and 0.1 Å, respectively. For cascade collision simulations, the typical PKA energy ranges from ∼1 keV to 100 keV, although lower or higher energies can also be considered depending on the studied irradiation scenarios. For ion irradiation studies, the energy transferred from the incident ion to the PKA carbon often exceeds 0.2 keV. Therefore, it is necessary to correct the short-range interactions when performing these irradiation simulations.

**Figure 7:**
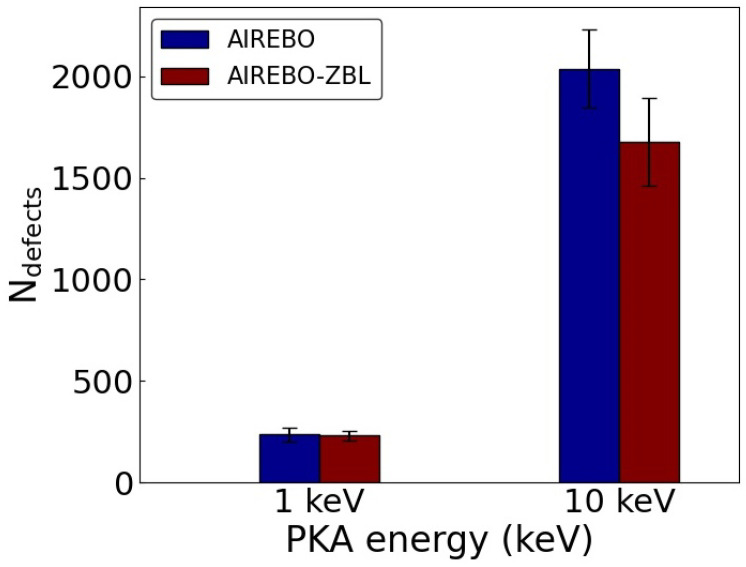
Number of atoms in the defective regions from cascade collisions of graphite with PKA energies of 1 keV and 10 keV using the AIREBO and AIREBO-ZBL potentials.

Cascade simulations were performed in graphite with PKA energies of 1 keV and 10 keV. The initial structural optimization shows that the closest C-C distances are 1.3958 Å and 1.3960 Å for the AIREBO and AIREBO-ZBL potential, respectively, with an interlayer distance of 3.358 Å for both. This result is consistent with Ref. [27], and the difference in lattice parameters is small between the two potentials. Figure 7 shows the number of atoms in the defective regions after the cascade collisions. At a PKA energy of 1 keV, the numbers of defects are 234.9 ± 34.9 and 229.9 ± 25.1 for the AIREBO and AIREBO-ZBL potential, respectively, indicating a minimal difference between the two. However, due to the difference in collision dynamics, a significant difference is observed at 10 keV. The numbers of defects are 2034.3 ± 193.2 and 1660.5 ± 191.8 for AIREBO and AIREBO-ZBL, respectively, with the difference amounting to approximately two standard deviations. The number of stabilized defects differs by 21%, with a higher number of defects generated by the original AIREBO potential.

**Figure 8:**
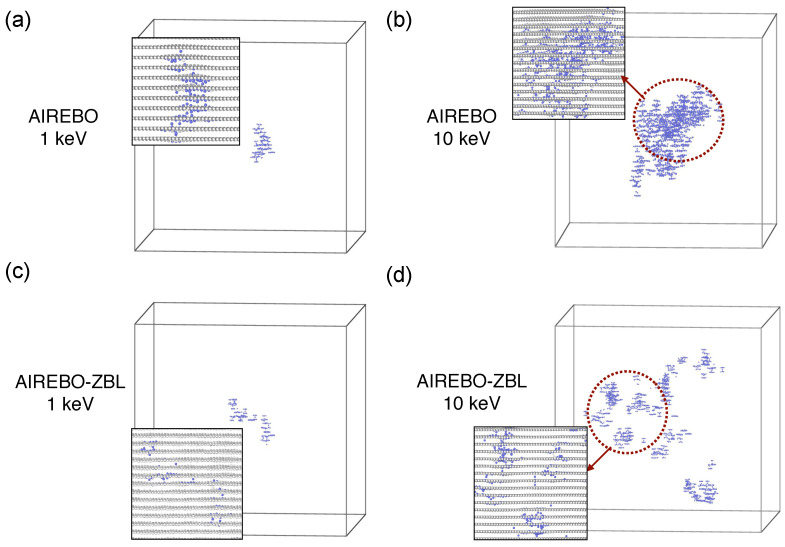
Morphology of the cascade-induced defective regions in graphite with the (**a**,**b**) AIREBO and (**c**,**d**) AIREBO-ZBL potential at 1 keV and 10 keV.

**Figure 9:**
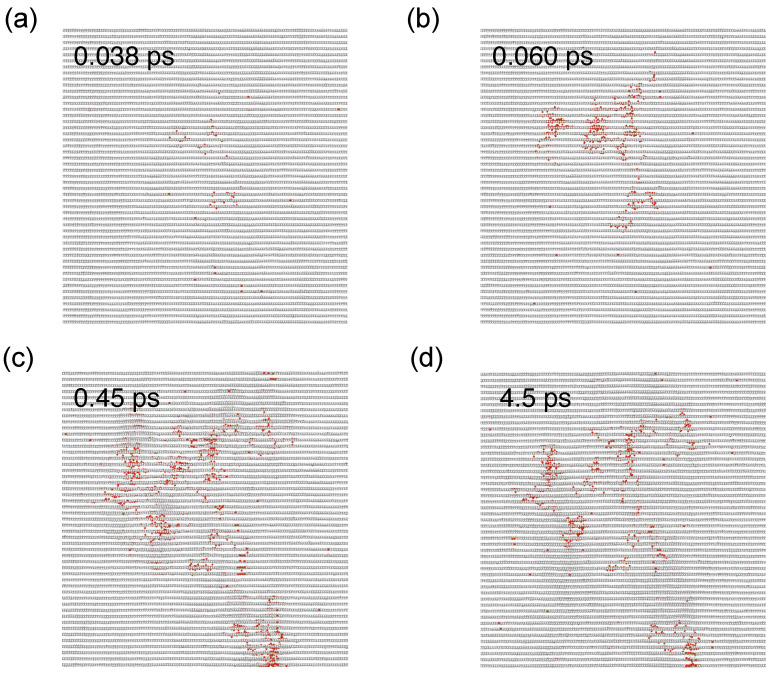
Representative cascade-induced defect evolution with the AIREBO-ZBL potential with a PKA energy of 10 keV. Red atoms correspond to atoms that are not properly bonded.

Figure 8 displays the representative spatial distributions of defects. Although the difference in defect morphology is less discernible at 1 keV, it becomes more pronounced at 10 keV. The defects are spatially more concentrated with the AIREBO potential, whereas they are more dispersed with the AIREBO-ZBL potential with a higher occurrence probability of small sub-cascades. At a PKA energy of 1 keV and a direction of 〈111〉, the distances between carbon atoms do not entirely fall within the short-range regime, where the interaction energies between the two potentials diverge. However, the significance of the short-range interaction description becomes evident at 10 keV. An important consequence of the weaker short-range interactions in AIREBO-ZBL is that the the energy lost per distance by the PKA is reduced when the atomic distance is close. This applies equally to secondary knock-on atoms and other atoms gaining kinetic energy. Therefore, the energy transfer is dispersed in a larger volume, leading to the generation of small sub-cascades. The time evolution of defect production during the initial damage stage is shown in Figure 9 for the AIREBO-ZBL potential with a PKA energy of 10 keV. It is evident that cascade collisions are more distributed, characterized by the occurrence of numerous small sub-cascades. Compared to the original AIREBO potential, a greater proportion of the initial kinetic energy goes into atomic thermal vibration within this expanded cascade volume without producing atomic displacement, resulting in a lower number of defective atoms. This again shows the critical role of accurate short-range interactions in irradiation simulations. In all cases, the defective regions comprise interstitial atoms, vacancies, and structural reordering. Interstitial atoms are predominantly located in the interlayer regions, leaving vacancies within the graphite layers. Local defect structures that differ from the standard six-atom carbon rings are observed due to atom displacement and subsequent reordering. We observe that at 10 keV with the AIREBO-ZBL potential, the lower energy loss of the PKA results in a longer trajectory, increasing the likelihood of traversing periodic boundaries. Given the current simulation box size, the chance of overlapping defect regions due to multiple PKA passages remains low. Nonetheless, a larger simulation box is recommended, provided computational resources permit. The Tersoff/ZBL potential was also used to simulate the cascade collisions in graphite [49], showing similar sub-cascade effects and dispersed defective regions. We also note that for performing cascade collision simulations, a high-index direction with less-dense atomic packing is typically chosen to represent the statistically average collision effects. In the particular case of head-on collisions, the minimum achievable C-C distance at 1 keV is also small (see Figure 6). Therefore, a noticeable difference in collision dynamics will also be anticipated.

For applications related to ion modification and defect engineering, the displacement cascade simulation results for defect generation probability, defect structures, and defect types are important for optimizing irradiation conditions. In addition, the results from the primary damage stage are crucial for understanding the subsequent evolution of defect structures. It is shown from this work that incorporating the ZBL short-range description is essential for determining the number, spatial distribution, and types of irradiation-induced defects. An ideal incorporation of short-range interactions should be performed during the initial potential development. Furthermore, calculations based on all-electron density functional theory can serve as input for a more accurate determination of atomic interactions within this distance range [50]. However, these methods require a deep understanding of potential fitting or require extensive computational work. The proposed method offers a convenient approach to correct the short-range behavior. To further improve accuracy at short and intermediate interatomic distances, input from a high-level physical description or calculation methodology is required.

## 4. Conclusions

A smooth connection of the ZBL potential with the AIREBO potential was demonstrated with a Fermi-like switching function to accurately describe the short-range interactions. The effects of ZBL integration on total potential energy and force, as well as different AIREBO energy components (REBO, LJ, and torsional energy), were critically examined across different structural configurations. The REBO component significantly influences the total energy profile, notably in the repulsive regime, whereas the LJ energy predominantly describes interactions at larger distances to account for intermolecular interactions. The short-range repulsive energy from the torsional component can be strong under certain circumstances, resulting in stiffer repulsive force than those from the screened Coulomb interaction. For different carbon structures, it is crucial to evaluate the behavior of various energy components at close interatomic distances to ensure an accurate representation of short-range interactions. Dynamics simulations revealed that the ZBL correction starts to significantly affect the collision process when the carbon kinetic energy is at the keV level. Cascade simulations in graphite also showed that the inclusion of the ZBL potential has a significant influence on the quantity and morphology of the cascade-induced defects. It has been demonstrated that an accurate description of short-range interactions is crucial for studying the effects of irradiation damage in carbon-based materials with high-energy ions using the AIREBO potential.

## Data Availability

The original contributions presented in the study are included in the article; further inquiries can be directed to the corresponding author(s).

## Abbreviations

The following abbreviations are used in this manuscript:

AIREBO	adaptive intermolecular reactive empirical bond-order
LAMMPS	large-scale atomic/molecular massively parallel simulator
LJ	Lennard-Jones
MD	molecular dynamics
PKA	primary knock-on atom
REBO	reactive empirical bond-order
ZBL	Ziegler-Biersack-Littmark
